# Restricted Mourning: Impact of the COVID-19 Pandemic on Funeral Services, Grief Rituals, and Prolonged Grief Symptoms

**DOI:** 10.3389/fpsyt.2022.878818

**Published:** 2022-05-27

**Authors:** Huibertha B. Mitima-Verloop, Trudy T. M. Mooren, Maria E. Kritikou, Paul A. Boelen

**Affiliations:** ^1^Department of Clinical Psychology, Utrecht University, Utrecht, Netherlands; ^2^ARQ National Psychotrauma Centre, Diemen, Netherlands; ^3^IDI Children and Adolescents Therapeutic Centre, Corfu, Greece

**Keywords:** COVID-19, bereavement, funeral, rituals, prolonged grief, pandemic, death

## Abstract

**Background:**

The COVID-19 pandemic has put various restrictions on grief rituals. Literature suggests that the restrictions on funerals and grief rituals may increase the chance of developing symptoms of prolonged grief (PG). In this study, we explored the possible impact of the pandemic on aspects of the funeral and grief rituals and examined their relationship with PG symptoms.

**Method:**

Bereaved individuals from different countries, who lost a loved one in the year prior to the pandemic (*n* = 50) or during the pandemic (*n* = 182), filled in an online questionnaire, including a rating of the impact of COVID-19 restrictions, five aspects of the funeral service, five aspects of grief rituals, and a measurement for PG symptoms.

**Results:**

Participants bereaved during the pandemic rated the impact of the restrictions on the experience of the funeral and grief rituals as negative. Nevertheless, no differences were found in attendance and evaluation of the funeral and grief rituals for people bereaved prior to vs. during the pandemic. Attendance and evaluation of the funeral services were related to levels of PG symptoms, whereas the performance and helpfulness of grief rituals were not related to these symptoms. Although not related to PG symptoms, half of the participants used helpful alternative rituals to cope with their loss.

**Discussion:**

Our study suggests that bereaved people respond resiliently to the COVID-19 pandemic, for example by creating alternative rituals to cope with their loss. Furthermore, it stresses the importance of looking beyond symptom levels when studying the importance of funeral and grief rituals.

## Introduction

Varying across countries and time, the recent COVID-19 pandemic has put various restrictions on engagement in grief rituals, such as not being able to perform religious rites at the bedside, online meetings with the funeral director, limited or no attendees during the funeral service, distance and no physical contact or not being allowed to visit the graveyard or come together for prayers (1). A bereaved individual described the death of a loved one during the pandemic as “a naked death to our culture”, because of the absence of grief rituals (2).

Funeral directors have expressed concerns that the absence of rituals could hold “frightening” mental health consequences for bereaved (1). Some bereavement scholars have also raised concerns about the effect that restricted or even absent funerals and grief rituals may have, such as a negative impact on the grieving process and adaptation, and increased chance of developing symptoms of prolonged grief (PG) (3–6). PG symptoms include intense reactions of grief that differ from the social, cultural, and religious norms and persist for a period longer than 6 months after the loss, causing impairment in functioning (7). The time criterion needs to be considered carefully to avoid pathologizing all bereaved individuals. Nevertheless, there are indications that increased PG symptoms early after the loss can predict higher levels of PG 6 month later, which is important to take into account (8).

Empirical findings obtained prior to the pandemic are inconsistent about the relationship between funerals, grief rituals, and grieving reactions. A recent systematic review of quantitative and qualitative studies conducted prior to the pandemic, revealed that most quantitative studies did not find a relationship between funeral practices, such as attendance of the funeral or evaluation of the funeral and grieving reactions (9). Some studies indicated effects for specific aspects of the funeral, such as experiencing the funeral as comforting, or being involved in planning of the funeral, being related to better grief adjustment. And occurrence of adverse events during the funeral being associated with severe grief (10). Two quantitative studies conducted after this review, but prior to the pandemic, did not find a relationship between satisfaction with cremation arrangements or funerals and grief responses either (11, 12). Mitima-Verloop et al. (13) studied the impact of grief rituals performed after the funeral service longitudinally and concluded that the number of grief rituals that people engaged in was not associated with changes in PG symptoms over time. Based on the qualitative studies in their review, Burrell and Selman (9) conclude that the benefit of rituals after bereavement depends on the ability of bereaved people to shape rituals in meaningful ways. Thus, they argue that COVID-19 pandemic restrictions do not necessarily affect grieving processes negatively.

A recurring issue in studies that are conducted prior to the COVID-19 pandemic is the limited variety in the nature, attendance, and evaluation of rituals; most people do attend a funeral or engage in grief rituals and evaluate these positively (13, 14). Moreover, these studies all had participants who made a voluntary decision about participating in a funeral or grief rituals, and were not restricted by circumstances or legislations by governments, such as during a pandemic.

The restrictions during the COVID-19 pandemic offer a new situation to study the importance of grief rituals among bereaved with probably greater diversity in nature, attendance, and evaluation of experiences. Previous studies provide some indications that particular restrictions can negatively impact grief responses (15). For example, a restricted number of people allowed at a funeral service potentially increases family conflict which may lead to adverse events during the funeral, a risk factor for intense grief reactions (1, 10, 11). Thus far, little consideration has been given to possible positive aspects of the COVID-19 restrictions (4).

To date, a few studies have been conducted during the COVID-19 pandemic investigating the association between characteristics of the funeral, grief rituals and grief responses. Menichetti Delor et al. (2) performed qualitative analyses of phone calls with 246 families bereaved due to COVID-19; they reported that mourners expressed a need to perform grief rituals, to find meaning and symbolic ways to say the last goodbye, and to express emotions to cope with the loss. In another qualitative study, based on digital media reports in Brazil, the restrictions on funeral rituals were considered to be a “traumatic experience”, causing feelings of disbelief and indignation (16). Hamid and Jahangir (17) concluded, based on 17 interviews with Muslims from Kashmir, that the inability to perform rituals added a layer to the grief, thus affecting the grieving process and overall wellbeing of those affected. In a cross-sectional study among 31 caregivers bereaved due to COVID-19, Bovero et al. (18) found that having the opportunity to attend the funeral was related to absence of complicated grief. Neimeyer and Lee (19) studied a group of 831 United States citizens, bereaved due to COVID-19 and identified several factors related to the pandemic that predispose grief outcomes. Higher endorsement of two items, “feeling upset that the deceased was not given the proper burial or memorial service” and “feeling upset about not being able to say goodbye to the deceased properly” was associated with functional impairment and dysfunctional grief. In contrast, a quantitative study among 114 Turkish bereaved individuals who lost a loved one during the pandemic did not find a relationship between attendance of the funeral or performance of grief rituals and grief reactions (20).

Taken together, most quantitative studies conducted prior to the pandemic demonstrated limited to no association between engagement in and evaluation of grief rituals and grief reactions over time. However, restrictions due to COVID-19 pandemic create a new situation, in which a few studies revealed contradictory results. Therefore, it is worthwhile to investigate the possible impact of the pandemic on various aspects of funeral services and grief rituals, and how this is associated with distressing grief reactions, including symptoms of PG. This may provide valuable information for professionals working with bereaved people. Accordingly, we conducted a cross-sectional survey study in a heterogenous convenience sample of bereaved people, enrolled in different countries.

The first aim of our study was to gain insight in the possible impact of COVID-19 pandemic on various aspects of the funeral service and grief rituals in different countries. It was hypothesized that participants would report a negative impact of the pandemic on their experience of the funeral and grief rituals. By more objective measures, it was hypothesized that individuals who suffered a loss during the pandemic would attend funerals less often, would perform less collective and individual rituals, would evaluate funerals as less positive, and the rituals they would perform as less helpful, compared to participants who lost their loved one prior to the pandemic. In addition, it was expected that participants would perform alternative rituals during COVID-19 pandemic. Our second aim was to examine the relationship between various aspects of the funeral service and grief rituals and the intensity of PG symptoms. Based on previous empirical studies, it was hypothesized that the performance and evaluation of the funeral service and grief rituals would be somewhat, albeit not very strongly, associated with the intensity of PG symptoms.

## Methods

### Design and Procedures

A cross-sectional study was conducted using online questionnaires that were distributed between November 2020 and December 2021. Ethical approval was obtained from the Ethics Review Board of the Faculty of Social and Behavioral Sciences of Utrecht University (FETC, 20-0221; 21-2009). The survey was made available in several languages, namely English, Greek, Spanish, German and Turkish. Dependent on availability, validated translations of questionnaires were used and remaining items were translated using forward/backward translation procedures. Bereaved individuals who lost a loved one since 2019 from various, mostly European, countries were invited to participate in the study. They were recruited via convenience sampling methods, such as announcements on social media groups for bereaved individuals, newsletters of funeral organizations, and researchers' personal network. All participants received an information letter and signed informed consent.

### Participants

A total of 251 participants completed the online survey. Participants who were bereaved before 2019 (*n* = 4), with unknown date of the loss (*n* = 12), or no complete questionnaires (*n* = 3) were excluded, leaving *N* = 232 participants for the analyses. Their age varied from 18 to 87 (*M* = 37.35, *SD* = 14.33) years. The number of days between the death of the loved one and completion of the questionnaire ranged from 1 through 1,017 days (33 months) (*M* = 274.50, *SD* = 205.01 days). Table 1 shows additional demographic and loss-related characteristics, for people bereaved before and during the pandemic.

**Table 1:** Demographics and loss-related characteristics of the sample and group differences (*n* = 232).

**Characteristic**		**Pandemic**	**Pre-pandemic**	**Test**
		*M* (*SD*)		*t-test*
Age		37.88 (14.16)	35.46 (14.92)	−1.06
Time since loss (in days)		218 (173.43)	480 (178.87)	9.41**
		*n* (%)		χ^2^
Gender	Male	30 (16.6)	8 (16.0)	0.01
	Female	150 (82.9)	42 (84.0)	
Education	Lower than University	78 (42.9)	9 (18.0)	10.51**
	University or higher	103 (56.6)	41 (82.0)	
Country of origin*	Western countries	124 (68.1)	42 (84.0)	4.85
	Non-western countries	58 (31.9)	8 (16.0)	
Religion	Christian	102 (56.1)	25 (50.0)	3.76
	Muslim	12 (6.6)	3 (6.0)	
	Atheist	23 (12.6)	12 (24.0)	
	Other (e.g., spiritual)	42 (23.1)	10 (20.0)	
Deceased	Close relative (i.e., partner, child, brother/sister, parent)	94 (52.2)	22 (44.0)	0.99
	Other relative/friend	87 (47.8)	28 (56.0)	
Cause of death	COVID-19	49 (26.9)	3 (6.0)	12.81**
	Illness (e.g., cancer)	37 (20.3)	17 (34.0)	
	Natural death (e.g., old age)	27 (14.8)	12 (24.0)	
	Other (e.g., unexpected medical causes, accident)	69 (37.9)	18 (36.0)	

*Participants were born in 32 different countries. Western countries were mostly represented by Greece (n = 55), Germany (n = 47), United Kingdom (n = 26) and Spain (n = 15). Non-western countries were mostly represented by Turkey (n = 31) and Mexico (n = 19).

**p < 0.01.

### Measures

Socio-demographic variables registered included (i) gender, (ii) age, (iii) level of education, (iv) country of birth, and (v) religious affiliation. Loss-related characteristics included (vi) relationship to the deceased, (vii) cause of death and (viii) date of the loss. A dichotomous variable was created for time of loss, with participants who experienced loss prior to the pandemic (between January 1st 2019 and March 10th 2020) coded as 0 and those who experienced loss during the pandemic (since March 11th 2020) coded as 1. March 11th 2020 was the date the World Health Organization (WHO) declared COVID-19 was a pandemic (21).

PG symptoms were measured with the 18-item Traumatic Grief Inventory self-report version [TGI-SR; (22)]. Participants rated how often they experienced 18 putative markers of PG in the past month (e.g., “I had trouble to accept the loss”) on 5-point scales (1 = never to 5 = always). The items of the questionnaire are in line with the DSM-5-TR and ICD-11 criteria of prolonged grief disorder (PGD), with a cutoff score of ≥61 indicating probable PGD. Research has shown good psychometric properties of the scale (23). Cronbach's alpha for the total scale was 0.94 in our study.

Five aspects of the funeral service were measured, namely (i) funeral attendance, (ii) funeral evaluation, (iii) comfort, (iv) planning, and (v) adverse events. (i) To rate funeral attendance, participants were instructed to score the item “Did you attend the funeral of your loved one?” with three possible options, namely “physical attendance”, “I did not attend” or “other (such as via livestream/online)”. (ii) Evaluation of the funeral was measured using the general evaluation scale of the Funeral Evaluation Questionnaire [FEQ; (13)]. This scale consists of four items, namely “I have been able to say good-bye to my loved one in the best way that was possible”, “The way in which the period around the funeral was organized, was important in processing the loss”, “I experienced the funeral as sad but positive”, and “The good-bye went exactly as I imagined it”. Items were scored on 5-point scales (1 = not at all to 5 = very much applicable). Cronbach's alpha for this scale in the present study was 0.80. The last three aspects were added based on the study of Gamino et al. (10), concerning possible factors that influence grief responses, namely (iii) comfort (i.e., “Do you describe the funeral of your loved one as comforting?”), (iv) planning (i.e., “Did you participate in the planning of the funeral?”), and (v) adverse events (i.e. “Were there any distressing or adverse events in connection to the funeral and if yes, how distressing were these events?”). Participants rated to what extent items applied to them on 5-point scales (1 = not at all to 5 = very much applicable).

Five aspects of grief rituals were measured, namely (i) performance of collective rituals, (ii) performance of individual rituals, (iii) helpfulness of collective rituals, (iv) helpfulness of individual rituals, and (v) helpful alternative rituals, based on the study of Mitima-Verloop et al. (13). Participants were instructed to score the item “What grief rituals or activities did you perform?” by selecting yes (scored as 1) or no (scored as 0) for “individual grief rituals in memory of the deceased (carried out alone)” and for “collective grief rituals (carried out with other people)”. When “yes” was selected, participants scored the item “How helpful were these activities in general?” for individual and/or collective grief rituals, on 5-point scales (1 = very unhelpful to 5 = very helpful). (v) Participants rated the statement “I found helpful alternative grief rituals to perform” on a 5-point scale (1 = not at all to 5 = very much).

The impact of COVID-19 pandemic on the experience of the funeral and grief rituals was measured using two self-constructed items, namely “Did the restrictions due to COVID-19 have an impact on the experience of (i) the funeral and (ii) grief rituals or activities after the funeral?” Both questions were scored on a 3-point Likert scale with 1 = very negative impact, 2 = negative impact and 3 = no negative impact. Because a pre-pandemic loss might have happened shortly before the date of declaring the pandemic, and engagement in grief rituals often takes place in the months to years after a loss, these questions were answered by all participants. In addition, participants were given the opportunity to explain their answer, with an open ended question. This question was not answered by all participants; some answers were quoted to illustrate findings from the quantitative analyses (see Discussion section).

### Statistical Analyses

Statistical analyses were performed using SPSS 27.0 (24). Two participants had one missing value on the TGI-SR questionnaire which were replaced using person mean imputation (25). To address our first aim, we compared participants bereaved before and during the pandemic, in terms of individual characteristics, loss-related variables, aspects of the funeral, and aspects of grief rituals. To this end, descriptive statistics, independent samples *t*-tests, and Pearson's chi-square tests were used. To correct for multiple testing, a significance level of *p* < 0.01 was applied. Regarding our second aim, we compared the groups pre-pandemic and during pandemic on PG symptoms, using descriptive statistics and an analysis of covariance (ANCOVA) to control for time since loss, cause of death and relationship to the deceased. Pearson correlations and point biserial correlation coefficients were calculated to evaluate associations between aspects of the funeral, aspects of grief rituals, and PG symptoms.

## Results

### The Impact of COVID-19 on Aspects of Funeral Services and Grief Rituals

Bereaved individuals who lost their loved one during the pandemic (*n* = 182) were compared to participants who lost their loved one prior to the pandemic (*n* = 50). Differences were found between level of education, with more people with higher education in the pre-pandemic group (see Table 1). Logically, the time since loss was significantly higher pre-pandemic and there were less COVID-19 related deaths reported (although a COVID-19 related death was possible in the days before the WHO declaration of COVID-19 as a pandemic). No significant differences between other demographic variables or loss-related characteristics were found.

Table 2 presents group differences for aspects of the funeral and grief rituals. Participants bereaved during the pandemic rated the impact of the restrictions on their experience of the funeral on average as (very) negative. The impact of the restrictions on the experience of grief rituals was also negative for participants during the pandemic. These scores were significantly more negative compared to the participants pre-pandemic, both for the impact of the restrictions on the experience of the funeral and for the experience of grief rituals, with large effect sizes (respectively *d* = 0.75 and *d* = 0.80).

**Table 2:** Aspects of the funeral, grief rituals and group differences (*n* = 232).

**Characteristic**		**Score**	**Pandemic**	**Pre-pandemic**	**Test**
			*n* (%)		χ^2^
Aspects of the funeral	Attended funeral?	Yes	126 (69.2)	39 (78.0)	1.56
		No	48 (26.4)	8 (18.0)	
		Other	8 (4.3)	2 (4.0)	
			*M* (*SD*)		*t-test*
	General evaluation (FEQ)	1–20	11.68 (4.57)	12.90 (4.82)	1.47
	How comforting was funeral?	1–5	2.63 (1.25)	2.95 (1.47)	1.40
	Participated in planning of funeral?	1–5	3.20 (1.63)	3.00 (1.52)	−0.68
	How distressing were adverse events?	1–5	2.40 (1.60)	2.00 (1.43)	−1.44
	Did COVID-19 affect funeral?	1–3	1.79 (0.79)	2.69 (0.58)	7.44*
			*n* (%)		χ^2^
Aspects of grief rituals	Performed individual rituals?	Yes	92 (50.5)	26 (52.0)	0.03
		No	90 (49.5)	24 (48.0)	
	Performed collective rituals?	Yes	101 (55.5)	34 (68.0)	2.52
		No	81 (44.5)	16 (32.0)	
			*M* (*SD*)		*t-test*
	How helpful were individual rituals?	1–5	3.40 (1.05)	3.42 (1.17)	0.11
	How helpful were collective rituals?	1–5	3.33 (1.11)	3.21 (1.18)	−0.56
	Finding helpful alternative rituals?	1–5	2.40 (1.19)	1.77 (1.06)	−3.35*
	Did COVID-19 affect rituals?	1–3	2.04 (0.84)	2.55 (0.65)	3.97*

*FEQ, Funeral Evaluation Questionnaire.*

*p < 0.001.

Despite the restrictions during the pandemic, around 70% of the participants physically attended the funeral of their loved one, while 26.4% did not attend a funeral service. Pre-pandemic, the percentage of participants attending the funeral was higher, although this difference was not significant. No group differences were found in the general evaluation of the funeral, the level of comfort, the involvement in planning or the level of distress of adverse events connected to the funeral (see Table 2).

During the pandemic, half of the bereaved individuals participated in individual rituals and more than half in collective rituals. Both types of rituals were evaluated as somewhat to quite helpful. No group differences were found on ritual performance or helpfulness of the rituals (see Table 2). Almost half of the participants (44.6%) indicated that they found helpful alternative rituals to perform (somewhat to very much). Finding helpful alternative rituals was significantly higher among individuals bereaved during the pandemic.

### Associations Between Funeral Services, Grief Rituals and PG Symptoms

On average, participants had a total score of 49.26 (minimum = 18, maximum = 85, *SD* = 16.31) on the TGI-SR. People who lost their loved one during the pandemic (estimated *M* = 49.94) had similar symptom levels of PG compared to participants before the pandemic (estimated *M* = 46.19), when controlling for time since loss, cause of death (COVID-19 and unexpected death vs. other) and relationship to the deceased, *F*_(1, 225)_ = 1.95, *p* = 0.16. Cause of death and relationship to the deceased were significant covariates. In the whole sample, 61 participants (26.4%) scored above the cutoff score of 61 indicating probable PGD. Of the 146 participants who lost their loved one more than 6 months ago, which is the formal timing criterion for a diagnosis of PGD as per ICD-11 (7), 33 participants (22.4%) scored above the cutoff score.

Table 3 shows correlations between aspects of the funeral, grief rituals, and PG symptoms. Funeral attendance was positively associated with PG symptoms, indicating higher symptoms levels for participants who attended the funeral. General funeral evaluation and experiencing the funeral as comforting were negatively related to PG symptoms, indicating more positive evaluation and more comfort related to less PG symptoms. Experiencing adverse event(s) and participating in planning was both associated with higher PG symptoms. No associations were found between performance and helpfulness of individual and collective grief rituals or finding helpful alternative rituals and PG symptoms.

**Table 3:** Pearson correlations and point biserial correlations between aspects of the funeral service, grief rituals and PG symptoms.

**Characteristic**		***n***	**PG symptoms**
Aspects of the funeral	Attended funeral? (0 = no, 1 = yes)	221	*r*_pb_ = 0.18*
	General Evaluation (FEQ)	173	*r =* −0.20*
	How comforting was funeral?	172	*r =* −0.33*
	Participated in planning of funeral?	173	*r = 0*.31*
	How distressing were adverse events?	174	*r = 0*.27*
	Did COVID-19 affect funeral?	222	*r =* −0.15
Aspects of grief rituals	Performed individual rituals? (0 = no, 1 = yes)	231	*r*_pb_ = 0.07
	Performed collective rituals? (0 = no, 1 = yes)	231	*r*_pb_ = −0.06
	How helpful were individual rituals?	117	*r =* −0.15
	How helpful were collective rituals?	133	*r =* −0.13
	Finding helpful alternative rituals?	221	*r* = −0.02
	Did COVID-19 affect rituals?	219	*r =* −0.13

*PG, Prolonged Grief. Funeral attendance is taken as a dummy variable, in which the answer “other” was disregarded.*

*r, Pearson correlation, r_pb_, point biserial correlation.*

*p < 0.01.

## Discussion

The present study explored the possible impact of the COVID-19 pandemic on various aspects of the funeral and grief rituals in a divers sample of bereaved individuals from different countries. Furthermore, it was examined how these aspects were related to PG symptoms.

Results revealed that participants rated the impact of the pandemic on their experience of both the funeral service and post-funeral grief rituals as (very) negative. Despite this, no significant differences in various aspects of the funeral service and grief rituals, such as funeral attendance, funeral evaluation, and the performance and helpfulness of individual and collective rituals, were observed between participants bereaved before vs. during the pandemic. This is striking, given the numerous restrictions that were put on engagement in grief rituals in countries all over the world (1). Apparently, the negative impact that participants experienced did not directly indicate less attendance or a negative evaluation of the funeral and grief rituals they engaged in. The non-significant difference in funeral attendance might be due to the fact that, in relation to the duration of the pandemic, the period and number of countries in which intimate family members were not or hardly allowed at funerals was relatively short. Furthermore, it is possible that different factors were connected to the negative impact of the restrictions, which were not considered in our study. For example, literature points toward the lack of social support from more extended family and friends who were not able to participate in the funeral due to restrictions (26).

These results might also suggest that bereaved individuals acknowledged that the restrictions had a major negative impact on their experiences, but also appreciated the funeral service as positive because it was the best way possible at that time. It is a well-known aspect of human judgment to evaluate situations to a reference point, and not in isolation (27). In the comment section of our questionnaire, participants described for example how grateful they were for the minimal support they received, how they experienced more solidarity and support because of the difficult circumstances, and how individual rituals such as lightning a candle helped them to grief their loved one alone. This is reminiscent of prior observations that, when adversity strikes a large group, the shared experience can have positive effects (28). During the pandemic, participants were more able to find helpful alternative rituals to perform. This outcome is consistent with previous qualitative studies conducted during the pandemic, reporting how people were able to create alternative rituals and creative new strategies to remember and grieve their loved ones (1, 29). These findings connect well with Burrell and Selman's (9) conclusion, that the benefit of rituals depends on the ability of bereaved individuals to shape rituals in such a way that is meaningful to them.

A considerable number of participants reported high levels of PG symptoms, even 6 months post loss. Symptom levels of PG were especially high among those bereaved after an unexpected death (including COVID-19) and those having had a close relationship to the deceased. This corresponds to previous studies, underlining the concern that the pandemic will lead to a higher prevalence of grief disorders [e.g., (30)].

Different aspects of the funeral were related to PG symptoms. However, the directions were not always as expected, and all correlations were small. The positive association between funeral attendance and PG symptoms could be explained by the fact that during the pandemic, often only close relatives were allowed to physically attend the funeral, and a close relationship to the deceased is a common factor related to more intense grief (31). Interestingly, this result differs substantially from the study of Bovero et al. (18), which included a very specific sample of bereaved individuals due to COVID-19 in the first wave of the pandemic in Italy. The great diversity of restrictions across time and across countries should be taken into consideration while comparing studies. Being involved in planning of the funeral was also related to higher PG symptoms, likely because involvement overlaps with being in a closer relationship with the deceased. An alternative explanation could be that funerals have been planned without the support of others due to restrictions on social contact (26). Further studies could focus more on characteristics of those attending in relation to other aspects of the funeral such as comfort and funeral evaluation.

Although associations between aspects of the funeral and PG symptoms were weak, these findings are interesting as they differ from the results of the longitudinal study of Mitima-Verloop et al. (13). In contrast to aspects of the funeral, and in line with Mitima-Verloop et al. (13), engagement in collective and individual rituals, their helpfulness, as well as performing alternative rituals were not related to PG symptoms. Besides looking at symptom levels of grief, further studies should focus on other aspects to get a better understanding of the impact of grief rituals. For example, Becker et al. (12) showed that dissatisfaction with the funeral was related to higher costs spent on medical and welfare services. Ritual elements, gaining more attention in therapeutic grief interventions, serve different functions and intensions, and their impact should therefore be measured in a broad sense, including concepts such as meaning and recognition (32–34). In addition, future studies could investigate the importance of cultural and religious values in relation to COVID-19 and grief, as restrictions led to major discrepancies between religious rituals, values, and government regulations (35).

Several limitations of this study should be mentioned. First, the study included a heterogeneous sample from different countries and in different times during the pandemic. Therefore, the results may not all apply to specific groups under specific circumstances during the pandemic. Furthermore, because of the convenience sampling methods, there is a possibility of response bias (e.g., underrepresentation of individuals with intense grief reactions). Nevertheless, a strength of the present study is that it extends previous research by including various aspects of the funeral service and grief rituals. In addition, the unique situation created by the COVID-19 pandemic led to greater diversity in engagement and experience of the funeral and grief rituals and therefore stronger measurements.

In conclusion, despite the negative impact of restrictions due to COVID-19 that many bereaved experienced, this impact seemed limited on engagement in and evaluation of funerals and grief rituals during the entire period of the pandemic. Associations were found between negative funeral experiences and symptom levels of PG, although these associations were weak. Besides the negative aspects, COVID-19 restrictions may also bring some positive aspects, such as experiencing more close connections because of the difficult circumstances and the possibility to create meaningful alternative rituals. Our results underline the resilience of individuals and the importance to look beyond symptoms when studying the importance of funerals and grief rituals.

## Data Availability Statement

The raw data supporting the conclusions of this article will be made available by the authors, without undue reservation.

## Ethics Statement

The study was reviewed and approved by the Ethics Review Board of the Faculty of Social and Behavioral Sciences of Utrecht University. The participants provided their informed consent to participate in this study.

## Author Contributions

HM-V and PB conceptualized and designed the survey and study. MK was involved in data collection and data analyses. HM-V drafted the manuscript. PB and TM provided critical feedback to the draft manuscript. All authors reviewed and approved the final version of the manuscript.

## Conflict of Interest

The authors declare that the research was conducted in the absence of any commercial or financial relationships that could be construed as a potential conflict of interest. The handling editor declared a past co-authorship with one of the authors PB.

## Publisher's Note

All claims expressed in this article are solely those of the authors and do not necessarily represent those of their affiliated organizations, or those of the publisher, the editors and the reviewers. Any product that may be evaluated in this article, or claim that may be made by its manufacturer, is not guaranteed or endorsed by the publisher.
